# Integrated Analysis Reveals the Potential Significance of HDAC Family Genes in Lung Adenocarcinoma

**DOI:** 10.3389/fgene.2022.862977

**Published:** 2022-08-22

**Authors:** Congkuan Song, Weichen Lin, Heng Meng, Ning Li, Qing Geng

**Affiliations:** Department of Thoracic Surgery, Renmin Hospital of Wuhan University, Wuhan, China

**Keywords:** histone deacetylases (HDAC), lung adenocarcinoma, prognosis, immune, drug

## Abstract

Histone deacetylases comprise a family of 18 genes, and classical HDACs are a promising class of novel anticancer drug targets. However, to date, no systematic study has been comprehensive to reveal the potential significance of these 18 genes in lung adenocarcinoma (LUAD). Here, we used a systematic bioinformatics approach to comprehensively describe the biological characteristics of the HDACs in LUAD. Unsupervised consensus clustering was performed to identify LUAD molecular subtypes. The ssGSEA, CIBERSORT, MCP counter, and ESTIMATE algorithms were used to depict the tumor microenvironment (TME) landscape. The Cox proportional hazards model and LASSO regression analyses were used to construct the HDAC scoring system for evaluating the prognosis of individual tumors. In this study, three distinct HDAC-mediated molecular subtypes were determined, which were also related to different clinical outcomes and biological pathways. HDACsCluster-C subtype had lowest PD-L1/PD-1/CTLA4 expression and immune score. The constructed HDAC scoring system (HDACsScore) could be used as an independent predictor to assess patient prognosis and effectively identify patients with different prognosis. High- and low-HDACsScore groups presented distinct genetic features, immune infiltration, and biological processes. The high-HDACsScore group was more likely to benefit from immunotherapy, as well as from the application of common chemotherapeutic agents (cyclopamine, docetaxel, doxorubicin, gemcitabine, paclitaxel, and pyrimethamine). Overall, HDAC family genes play important roles in LUAD, and the three LUAD subtypes and the HDAC scoring system identified in this study would help enhance our perception of LUAD prognostic differences and provide important insights into the efficacy of immunotherapy and chemotherapy.

## Introduction

Histone acetylation is accomplished by histone acetyltransferases (HATs) and histone deacetylases (HDACs), controlling the transcription level of genes and playing a key role in structural modification of chromosomes and the regulation of gene expression (Kim et al., 2020; Weinberger et al., 2012; Xu et al., 2012). Of them, HDACs, as gene-silencing complexes, can inhibit gene expression through transcription factors such as E2F1 and can also eliminate the acetylation of nonhistones (Fang et al., 2020; Kim et al., 2019). At present, it has been found that there are four categories of HDACs, including 18 subtypes. Class I includes HDAC1, HDAC2, HDAC3, and HDAC8; class II includes HDAC4, HDAC5, HDAC6, HDAC7, HDAC9, and HDAC10; class III includes sirtuin (SIRT) 1, SIRT2, SIRT3, SIRT4, SIRT5, SIRT6, and SIRT7; and class IV includes HDAC11. HDAC overexpression promotes histone deacetylation, enhancing the interaction between histones and DNA, and thus inhibiting the transcriptional processes of related genes (Kim et al., 2016; Sanchez et al., 2020). HDACs are often overexpressed in tumor cells (Yang et al., 2014). Many studies (Li et al., 2020; Maiti et al., 2019; Torres-Adorno et al., 2017) have confirmed the involvement of HDACs in different stages of cancer. HDACs have become a popular target for antitumor drug development (Dokmanovic and Marks., 2005; Bolden et al., 2006; Li and Zhu, 2014).

Lung adenocarcinoma (LUAD), as one of the most common histological subtypes in non–small cell lung cancer (NSCLC), has complex heterogeneity (Raparia et al., 2013; Travis et al., 2005), posing great challenges for clinical treatment. Although the diagnosis and treatment of LUAD have made great progress in recent years, there is still room for great improvement in the long-term prognosis of patients. Previous studies (Li and Seto., 2016; Mithraprabhu et al., 2014) have shown that an increased expression of HDAC was observed in multiple solid tumors and was related to the poor prognosis of patients. Compared with normal lung cells, lung cancer cells have abnormal histone modification patterns (Li and Seto., 2016), which play a crucial role in lung carcinogenesis. The mechanisms by which HDAC regulates tumorigenesis and progression are complex and diverse, and it can regulate oncogenic cell signaling pathways both by inhibiting the expression levels of tumor suppressor genes and by modifying key molecules (Li and Seto., 2016). A full understanding of the important role of HDAC in lung cancer is of great significance for analyzing the mechanism of tumorigenesis, improving the possibility of clinical application of HDAC inhibitors and individualized treatment strategies for lung cancer. HDAC family genes are still poorly studied in LUAD. To date, no systematic study has been comprehensive to reveal the potential significance of these 18 HDACs in LUAD. Therefore, this study used a systematic bioinformatics approach to comprehensively describe the biological characteristics of the HDACs in the LUAD, performing unsupervised clustering based on HDACs to identify HDAC-mediated molecular subtypes, while constructing the HDAC scoring system to explore their regulatory relationship with the tumor.

## Materials and Methods

### Data Collection and Preprocessing

First, we searched for appropriate gene microarray containing transcriptomic data and clinical information for LUAD from the GEO database as well as publicly published literature, excluding datasets with sample sizes of less than 30 cases and lacking HDAC family genes (HDAC 1-11 and SIRT 1-7), and we finally included a six-gene microarray [GSE29013 (Xie et al., 2011), GSE30219 (Rousseaux et al., 2013), GSE31210 (Okayama et al., 2012; Yamauchi et al., 2012), GSE37745 (Botling et al., 2013; Goldmann et al., 2021; Jabs et al., 2017; Lohr et al., 2015), GSE50081 (Der et al., 2014), and GSE72094 (Schabath et al., 2016)] in this study. In addition, we also downloaded data on LUAD transcriptomes, somatic mutations, and clinical information from TCGA GDC (https://portal.gdc.cancer.gov/). In Supplementary Table S1, we summarize the number of samples and platform names of patients with LUAD from the abovementioned datasets. The CNV data were obtained from the UCSC Xena (http://xena.ucsc.edu/). For genes with multiple probe sets of signals, we averaged them to generate single-expression values. For different gene microarrays, we used the “"ComBat” algorithm of the “sva” package in R to further integrate into a metacohort to reduce batch effects resulting from nonbiotechnological bias (Irizarry et al., 2003; Johnson et al., 2007). Expression data before and after the removal of batches were analyzed by PCA with the base function “prcomp” of R.

### Unsupervised Consensus Clustering

By integrating into a metacohort from the abovementioned six-gene microarray, based on the expression of related genes, we applied unsupervised consensus clustering analysis to identify different molecular clusters and classify patients for further analysis. The “ConsensusClusterPlus” package in R (Wilkerson and Hayes., 2010) was used to perform the abovementioned steps and also perform 1,000 replicates to ensure stability of the classification.

### Functional and Pathway Enrichment Analysis

To investigate the differences in biological processes between different subgroups, GSVA enrichment analysis was performed using the “GSVA” R package (Hanzelmann et al., 2013). The gene set “c2. cp.kegg.v7.2. symbols” was derived from the MSigDB (http://www.gsea-msigdb.org/gsea/msigdb). The adjusted *p*-value of less than 0.05 was considered statistically significant. GO and KEGG enrichment analyses were also used to explore the activation of biological processes between groups. The associated genes were functionally annotated using the “clusterProfiler” R package (Yu et al., 2012), with a cutoff value of FDR <0.05.

### Characterization of the Tumor Microenvironment and Immune Landscape

To better characterize the tumor microenvironment (TME) and immune landscape in different subtypes, we used multiple algorithms, including ssGSEA (Barbie et al., 2009), CIBERSORT (Newman et al., 2015), and MCP counter (Becht et al., 2016), to quantify the abundance of each immune infiltrating cell. Meanwhile, the ESTIMATE algorithm (Becht et al., 2016) was used to impute the tumor purity, stromal score, and immune score.

### Establishment of an Evaluation System Associated With the HDAC Phenotypes

Univariate Cox analysis was performed on HDACsCluster phenotype-related genes, and then the genes with *p* < 0.05 were included in Lasso regression analysis for further dimensionality reduction. The filtered genes were subjected to multivariate Cox analysis (stepwise regression) to construct a scoring system for assessing patient outcomes. The calculation formula for HDACsScore was as follows: HDACsScore = Coef ***G1*** *Expression ***G1*** + Coef ***G2*** *Expression ***G2*** + ... + Coef ***Gn*** *Expression ***Gn*** *,* where *“*Expression ***Gn*** *”* is the expression of “gene ***n***”, and “Coef ***Gn”*** presented the coefficient of “gene ***n***”.

### Statistical Analysis

The “limma” package in R was used for gene differential expression analysis. The statistical difference between two groups was calculated using the Wilcoxon rank sum test. For comparisons of more than two groups, the Kruskal–Wallis test was used. Survival comparison between two or multiple groups was performed using the Kaplan–Meier method. We used the “pRRophetic” package in R (Geeleher et al., 2014) to impute the semi-inhibitory concentration (IC_50_) of the drugs to assess the sensitivity of patients to a given drug. A website named Tumor Immune Dysfunction and Exclusion (TIDE) (http://tide.dfci.harvard.edu) was applied to compute TIDE scores and assess patient sensitivity to immunotherapy (Jiang et al., 2018). The *p* < 0.05 was considered statistically significant. All data processing was carried out using R3.6.2 software.

## Results

### Biological Characterization of the HDAC Family Genes

**FIGURE 1:**
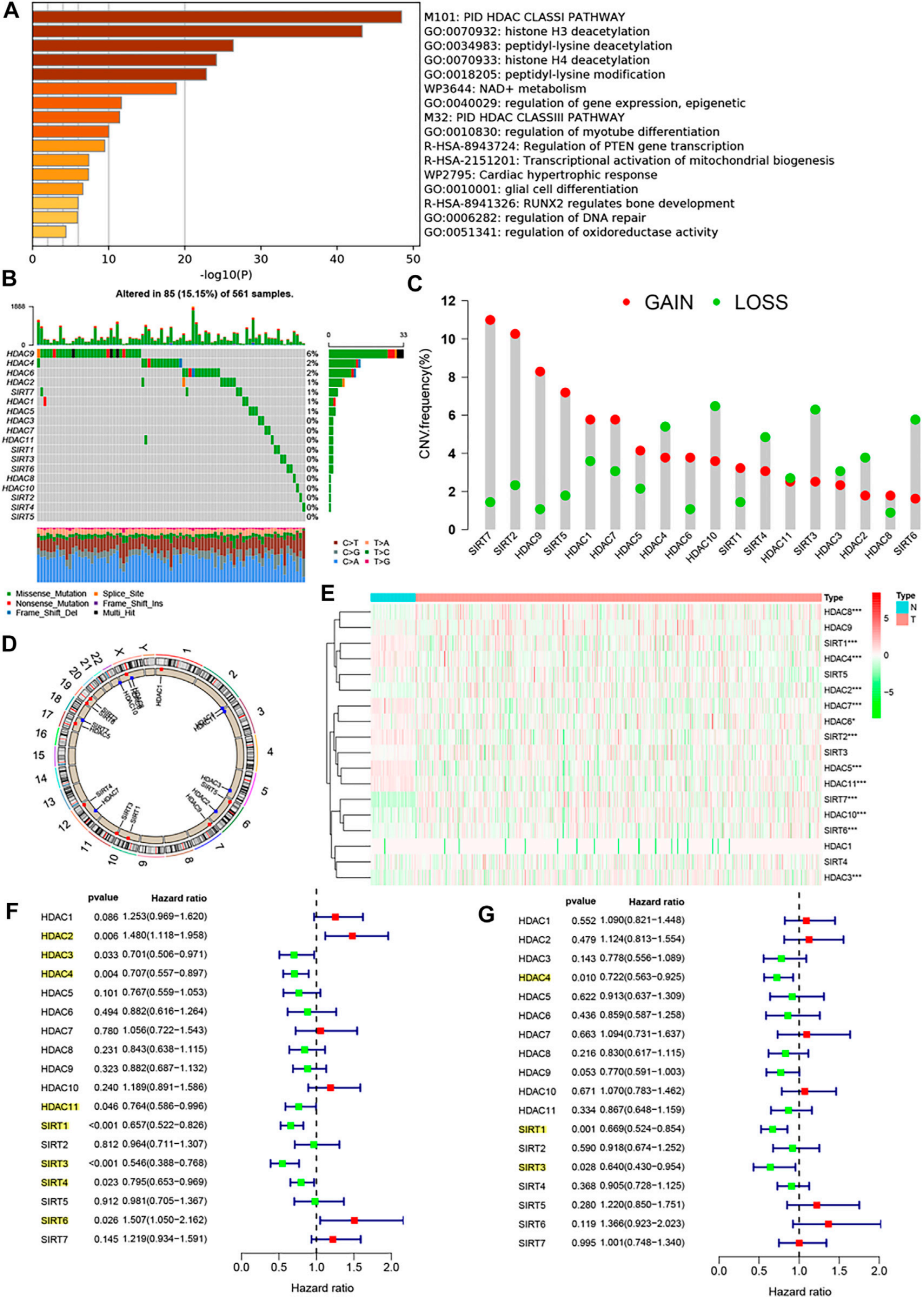
Biological characterization of the HDAC family genes. **(A)** Metascape analysis revealed the biological function of the 18 HDAC family genes. **(B)** Mutation frequency of 18 HDAC family genes in 561 LUAD patients from the TCGA−LUAD cohort. Each column represented individual patients. The upper barplot showed TMB, and the number on the right indicated the mutation frequency in each regulator. The right barplot showed the proportion of each variant type. The stacked barplot showed fraction of conversions in each sample. **(C)** CNV variation frequency of 18 HDAC family genes in the TCGA−LUAD cohort. The height of the column represented the alteration frequency. The deletion frequency, green dot; the amplification frequency, red dot. **(D)** Location of CNV alteration of 18 HDAC family genes on 23 chromosomes using the TCGA−LUAD cohort. **(E)** Expression of 18 HDAC family genes between lung normal tissues and LUAD tissues. The asterisks represented the statistical *p*-value (*p < 0.05; **p < 0.01; ***p < 0.001). **(F)** Univariate Cox analysis of 18 HDAC family genes in the TCGA−LUAD cohort. **(G)** Multivariate Cox analysis of 18 HDAC family genes in the TCGA−LUAD cohort.

By consulting the relevant reports, we obtained a total of 18 HDAC family genes, and they are HDAC1, HDAC2, HDAC3–HDAC11, SIRT1, SIRT2, and SIRT3–SIRT7. Subsequently, we revealed the biological function of the family genes using metascape analysis, as shown in Figure 1A, which were primarily involved in PID HDAC Class-I pathway, histone H3 and H4 deacetylation, peptidyl-lysine deacetylation, peptidyl-lysine modification, etc. We summarized the incidence of CNVs and somatic mutations of the 18 HDAC family genes in LUAD. Among 561 samples, 85 experienced mutations of HDAC family genes, with a frequency of 15.15%. Of these genes, HDAC9 exhibited the highest mutation frequency (6%) followed by HDAC4 and HDAC6 (2%), while HDAC3, HDAC7, HDAC10, HDAC11, as well as SIRT1-6 did not show any mutations in LUAD samples (Figure 1B). Considering the relatively higher mutation frequency of HDAC9, we further investigated whether genetic variations in HDAC9 could affect the expression of other HDAC genes. The results of the abovementioned analysis showed that this HDAC9 low-frequency mutation would promote HDAC2 expression and did not appear to affect the expression of other HDACs (Supplementary Figure S1). Further analysis of the 18 HDAC family genes revealed that CNV mutations were prevalent. SIRT7, SIRT2, HDAC9, SIRT5, HDAC1, HDAC7, HDAC5, HDAC6, SIRT1, and HDAC8 showed widespread CNV amplification. In contrast, HDAC4, HDAC10, SIRT4, HDAC11, SIRT3, HADC3, HDAC2, and SIRT6 had prevalent CNV deletions (Figure 1C). The locations of CNV alterations of the 18 HDAC family genes on chromosomes are shown in Figure 1D. To ascertain whether the abovementioned copy number variants affected the expression of HDAC family genes in LUAD patients, we performed correlation exploration using the Kruskal–Wallis test (Supplementary Figure S2) and investigated the transcriptomic levels of HDAC family genes between lung normal and LUAD samples in the TCGA–LUAD cohort (Figure 1E). The results revealed that the alterations of CNV could be the prominent factors resulting in perturbations on the HDAC family gene expression. Copy number amplification of these genes, such as HDAC1, HDAC4, HDAC5, HDAC6, HDAC7, HDAC10, SIRT2, SIRT4, SIRT5, SIRT6, and SIRT7, upregulated the expression of the corresponding genes, while their copy number deletion downregulated gene expression. Copy number amplification of these genes, such as HDAC8, HDAC9, and SIRT1, upregulated the expression of the corresponding genes, while their copy number deletion did not affect the gene expression. HDAC2 and HDAC3 expressions did not appear to be affected by copy number variation. Moreover, differential expression analysis of the abovementioned genes uncovered that the expressions of HDAC8, HDAC2, SIRT7, SIRT6, HDAC10, and HDAC3 were upregulated in LUAD tissues and the expressions of SIRT1, HDAC4, HDAC7, HDAC6, SIRT2, HDAC5, and HDAC11 were upregulated in lung normal tissues. From these, we observed that the copy number alterations of these genes did not cause differences in their expression between tumor and normal tissues.

**FIGURE 2:**
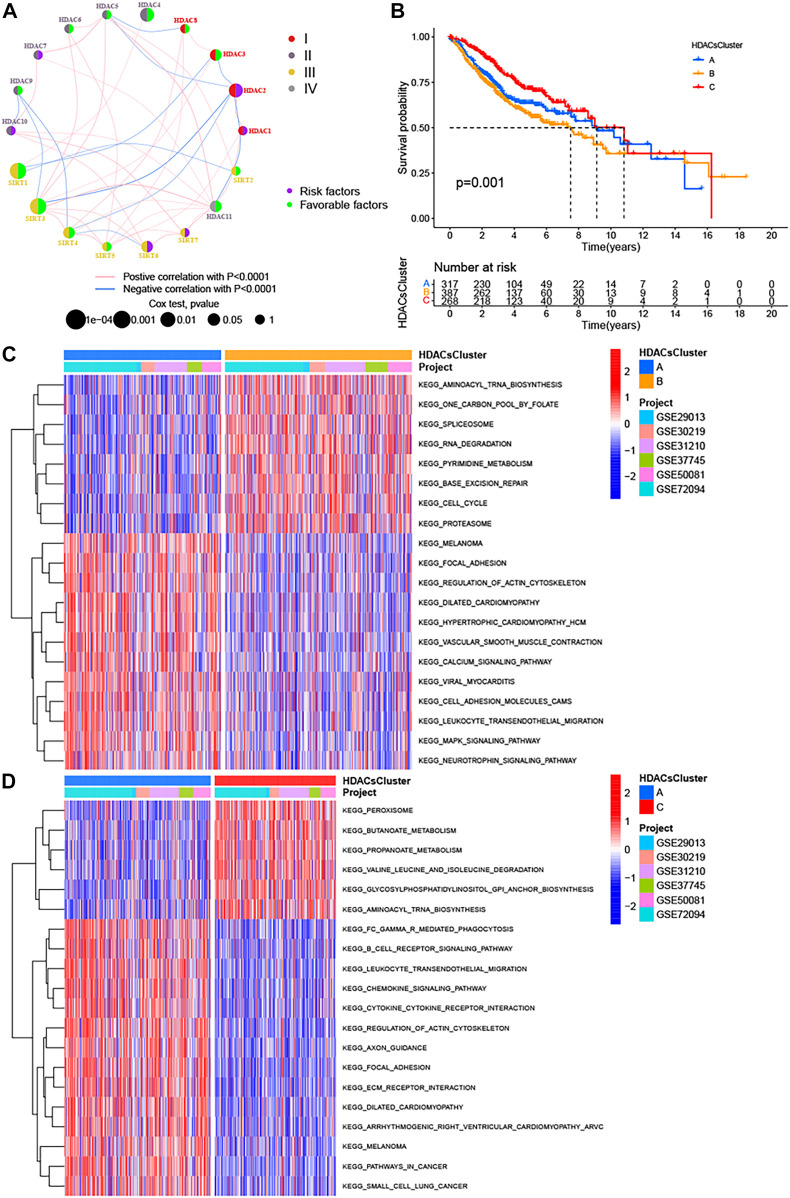
Three distinct LUAD subtypes and biological characteristics of each subtype. **(A)** Interaction between 18 HDAC family genes in LUAD. The circle size represented the effect of each regulator on the prognosis, and the range of values calculated by the log-rank test was *p* < 0.0001, *p* < 0.001, *p* < 0.01, *p* < 0.05, and *p* < 1, respectively. Left half of the circle: purple represents prognostic risk factors, and green represents prognostic favorable factors. Right half of the circle: the types of 18 HDAC family genes. The lines linking regulators showed their interactions, and thickness showed the correlation strength between regulators. Negative correlation was marked with blue and positive correlation with pink. **(B)** Kaplan−Meier survival analyses for the three distinct LUAD subtypes based on 972 patients from six GEO cohorts (GSE29013, GSE30219, GSE31210, GSE37745, GSE50081, and GSE72094) including 317 cases in HDACsCluster-A, 387 cases in HDACsCluster-B, and 268 cases in HDACsCluster-C. **(C–D)** GSVA enrichment analysis showing the activation states of biological pathways in distinct LUAD subtypes. The heat map was used to visualize these biological processes, and red represented activated pathways and blue represented inhibited pathways. The LUAD cohorts were used as sample annotations. **(C)** HDACsCluster-A vs HDACsCluster-B; **(D)** HDACsCluster-C vs HDACsCluster-A.

Increasing evidence has reported a close link between HDAC family genes and cancer; however, there is not still a more systematic study to investigate the impact of these 18 family genes in the prognosis of LUAD. Univariate Cox regression analysis and Kaplan–Meier survival analysis were used to ascertain the relationship between these genes and the prognosis of LUAD patients. The results showed that whether in the univariate Cox regression analysis (Figure 1F) or in the Kaplan–Meier survival analysis (Supplementary Figure S3), HDAC3, HDAC4, HDAC11, SIRT1, SIRT3, and SIRT4 could be considered protective factors and were significantly associated with prolonged overall survival, while HDAC2 and SIRT6 were recognized as risk factors. Subsequent multivariate Cox analysis revealed that HDAC4, SIRT1, SIRT3, and SIRT4 were independent good prognostic factors for LUAD patients (Figure 1G). The correlation between the expression of HDAC family genes and immune cell infiltration remains poorly explored, and our study showed that the vast majority of HDAC genes were negatively correlated with the vast majority of immune cells, whereas HDAC3, HDAC9, and SIRT2 showed the opposite trend (Supplementary Figure S4). In addition, the vast majority of HDAC genes also showed slight positive correlations with each other (Supplementary Figure S4 and Figure 2A).

The abovementioned analyses characterized the biological characteristics of HDAC family genes in LUAD from biological function, transcriptome expression pattern, genomic alteration, prognosis, and immune cell infiltration, and these results indicated the important roles of HDAC family genes in LUAD.

### Three Distinct LUAD Subtypes Were Identified Based on the Unsupervised Consensus Clustering of 18 HDAC Family Genes

Many studies (Raparia et al., 2013; Travis et al., 2005) have confirmed there is significant heterogeneity in LUAD, which also brings great challenges to the prognostic judgment and treatment decisions in LUAD patients. It is necessary to further determine the tumor subtypes with different characteristics to adopt precise strategies. Given the important roles of HDAC family genes in LUAD, we believed that they probably played critical roles in the formation of different tumor subtypes and were implicated in cancer pathogenesis and progression. Based on these hypotheses, we used the “ConsensusClusterPlus” package in R to classify patients with qualitatively different HDAC modification patterns based on the expression of 18 HDAC family genes, and three distinct tumor subtypes were eventually identified using unsupervised clustering (Supplementary Figure S5A), including 317 cases in subtype A, 387 cases in subtype B, and 268 cases in subtype C. We termed these subtypes as HDACsCluster A−C, among which HDACsCluster-C exhibited a prominent survival advantage, whereas HDACsCluster-B had the worst prognosis (Figure 2B). Additionally, we also noticed significant differences in the expression of HDAC family genes between distinct tumor subtypes. HDAC1, HDAC2, SIRT6, and SIRT7 were significantly elevated in the HDACsCluster-B subtype. HDAC9 and SIRT2 were markedly increased in the HDACsCluster-A subtype. Also, HDAC3, HDAC5, HDAC6, HDAC8, HDAC10, HDAC11, SIRT1, SIRT3, SIRT4, and SIRT5 were evidently increased in the HDACsCluster-C subtype (Supplementary Figure S5B). We could conclude that a vast number of HDAC family genes were upregulated in the HDACsCluster-C subtype and that most of these genes were prognostic-friendly genes (Supplementary Figure S3), which could explain why HDACsCluster-C subtype presented a best prognosis.

### The Biologic Pathways and TME Landscape in Distinct Tumor Subtypes

**FIGURE 3:**
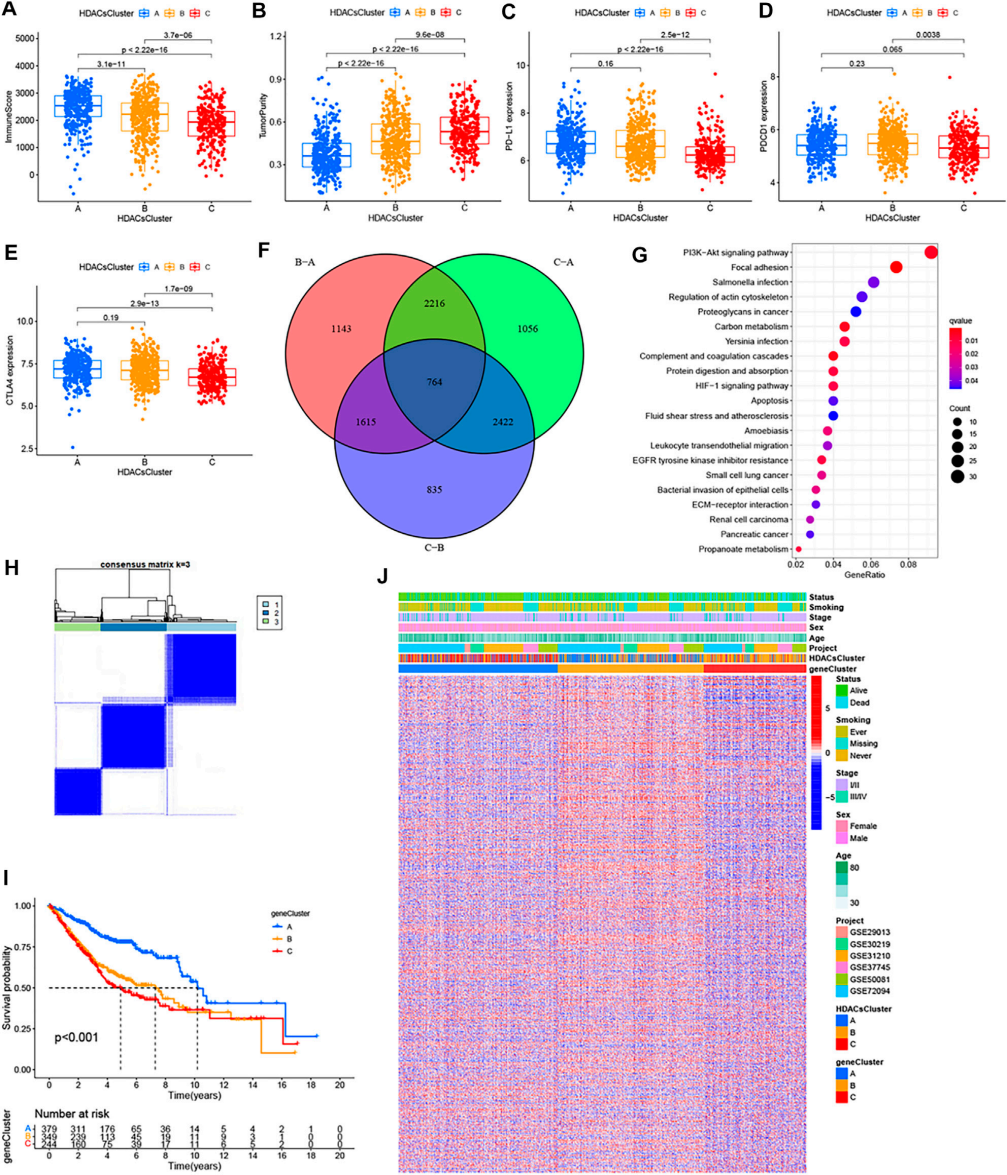
Immune landscapes among the three distinct molecular subtypes and HDAC phenotype–related reclustering. **(A)** Immune score, **(B)** tumor purity, **(C)** PD-L1 expression, **(D)** PDCD1 expression, and **(E)** CTLA-4 expression differences in three distinct molecular subtypes. **(F)** 764 HDAC phenotype–related differentially expressed genes (DEGs) between three distinct molecular subtypes were shown in the Venn diagram. **(G)** Functional annotation for phenotype-related DEGs associated with the prognosis using KEGG enrichment analysis. **(H)** Unsupervised clustering of the HDAC phenotype–related genes and consensus matrices for k = 3. **(I)** Survival curves of the HDAC phenotype–related gene signatures were estimated by the Kaplan−Meier plotter. **(J)** Unsupervised clustering of HDAC phenotype–related DEGs. The geneCluster, HDACsCluster, project, age, sex, stage, smoking status, and survival status were used as patient annotations. Red represented high expression of regulators and green represented low expression.

To explore the biological behaviors across these different subtypes, we performed GSVA enrichment analyses in the meta-GEO cohort. Supplementary Figure S6 reflects the PCA plots before and after the batch effect removal. As shown in Figures 2C,D; Supplementary Figure S5C, HDACsCluster-A presented the activation of important signals and pathways including leukocyte transendothelial migration, B cell receptor signaling pathway, and glycosphingolipid biosynthesis ganglio series. HDACsCluster-B was markedly enriched in aminoacyl tRNA biosynthesis, DNA replication, mismatch repair, homologous recombination, base excision repair etc, while HDACsCluster-C was prominently related to body material metabolism including butanoate metabolism; propanoate metabolism; valine, leucine, and isoleucine degradation; and aminoacyl tRNA biosynthesis. These results suggested significant differences in pathway activation across the three tumor subtypes. Subsequently, in the analysis of tumor immune cell infiltration, to our surprise, there were 19 immune cells (both immunosuppressive cells and immune-activated cells) with the highest infiltration levels in HDACsCluster-A (Supplementary Figure S5D). Consistent with this finding, ESTIMATE analysis also revealed HDACsCluster-A presented a highest immune score and lowest tumor purity (Figures 3A,B). Given that the expression of the immune checkpoints could somewhat reflect the therapeutic efficacy of patients against PD-1/L1 treatment, we also compared the expression levels of PD-L1, PD-1, and CTLA4 in three different clusters. We observed that compared with HDACsCluster-A and -B, HDACsCluster-C had lowest immune checkpoint expression (Figures 3C–E). This might somehow imply a poor response of HDACsCluster-C subtype to immunotherapy.

### HDACsCluster Phenotype-Related DEGs and Characteristics in LUAD

Although the consensus clustering algorithm based on HDAC family gene expression classified LUAD patients into three HDACsCluster phenotypes, the underlying molecular features and expression perturbations within these phenotypes were not well known. Thus, we further examined the potential HDAC-related transcriptional expression change across three subtypes in LUAD. The empirical Bayesian approach was applied to determine overlapping differentially expressed genes (DEGs) among the three subtypes. We determined 764 HDACsCluster phenotype-related DEGs using the limma package in R (Figure 3F). GO and KEGG enrichment analyses of these DEGs revealed that these genes were mainly involved in some vital biological processes and pathways, such as PI3K/Akt signaling pathway, focal adhesion, regulation of actin cytoskeleton, extracellular matrix organization, and collagen−containing extracellular matrix (Figure 3G; Supplementary Figure S7A). Subsequently, based on these genes, we once again performed the unsupervised clustering analysis and obtained three stable transcriptomic phenotypes (Figure 3H). These stratifications divided patients into three distinct subgroups (geneCluster-A, geneCluster-B, and geneCluster-C). Kaplan−Meier survival analysis revealed the patients in geneCluster-A showed the longest overall survival compared to the others (Figure 3I). The relationship between these three new clusters, HDACsCluster, clinical parameters, and the gene expression distribution was visualized as a heat map in Figure 3J, where we found the vast majority of the samples in HDACsCluster-C were corresponding to the geneCluster-A, the vast majority of the samples in HDACsCluster-B were corresponding to the geneCluster-C, and the vast majority of the samples in HDACsCluster-A were corresponding to the geneCluster-B. This relationship also corresponded one by one to their respective prognostic differences (Figure 2B, Figure 3I). The heat map in Figure 3J revealed that the vast majority of 764 HDACsClusters phenotype-related DEGs were also highly expressed in the geneCluster-B. Moreover, HDAC8, HDAC11, SIRT1, SIRT3, and SIRT4 were obviously highly expressed in geneCluster-A than in geneCluster-C/B, while HDAC1, HDAC2, HDAC10, SIRT6, and SIRT7 had the highest expression value in the geneCluster-C (Supplementary Figure S7B). While our results (Supplementary Figure S3) indicate that HDAC8, HDAC11, SIRT1, SIRT3, and SIRT4 were good prognostic factors and HDAC1, HDAC2, SIRT6, and SIRT7 were unfavorable prognostic factors, this could also explain why geneCluster-A had the best prognosis, while geneCluster-C presented a poor prognosis (Figure 3I), suggesting that HDAC family genes did play an important role in LUAD prognosis and that the three subtypes identified in this study had good risk stratification performance.

### Construction of the HDACsScore System

**FIGURE 4:**
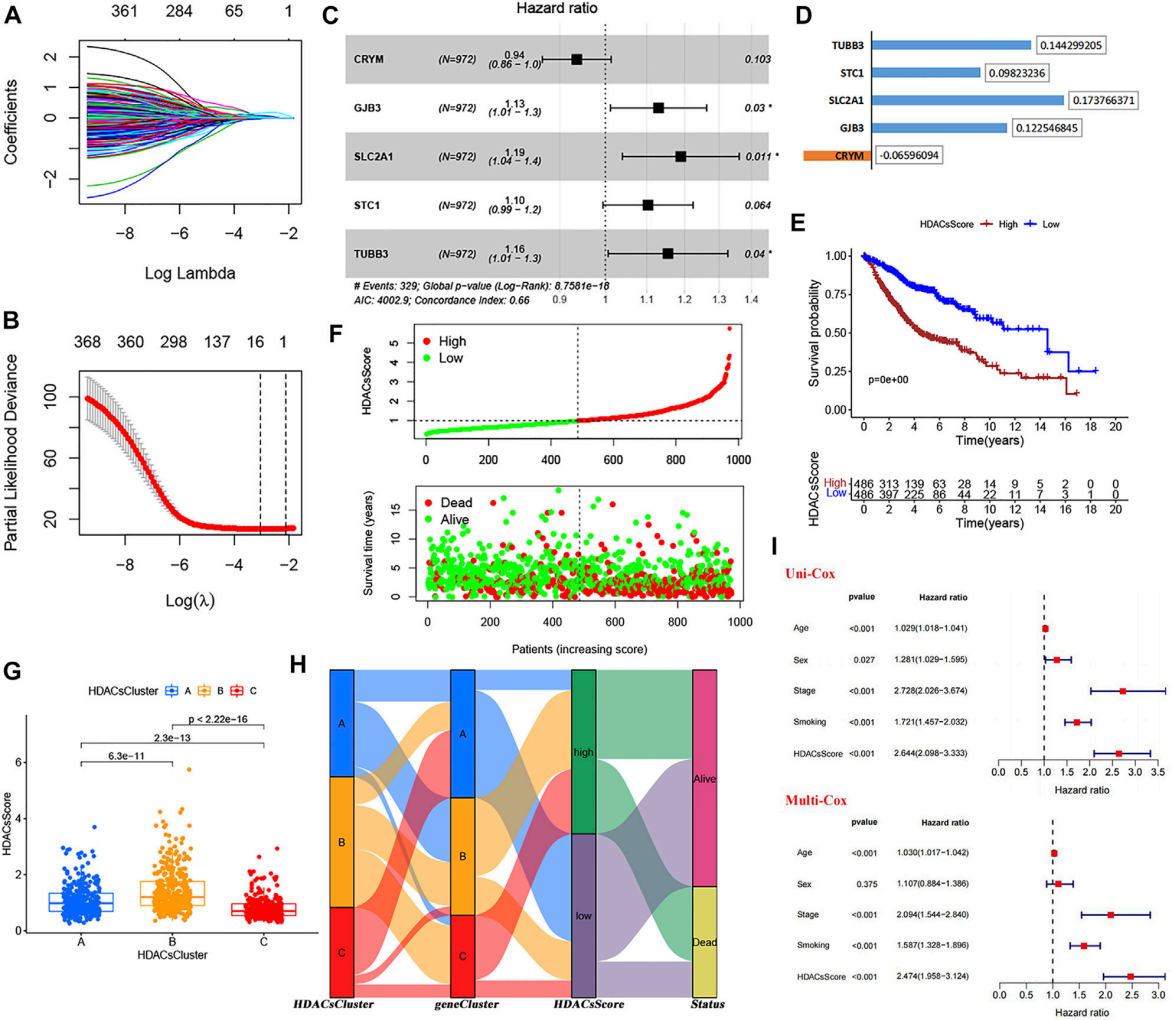
Construction and validation of the HDAC scoring system. **(A**−**B)** LASSO regression analysis of 371 prognosis-related DEGs to avoid the model overfitting. **(C)** Multivariate Cox analysis (stepwise regression) of the filtered genes in the meta-GEO cohort. **(D)** Correlation coefficient of the model genes. **(E)** Survival analyses for low- (486 cases) and high-(486 cases) HDACsScore groups using Kaplan−Meier curves. **(F)** HDACsScore and OS status distribution of the model in the meta-GEO cohort. **(G)** Correlations between HDACsScore and HDAC-mediated molecular subtypes (HDACsCluster). **(H)** Alluvial diagram showing the changes of HDACsClusters, geneCluster, HDACsScore, and OS status. **(I)** Univariate and multivariate Cox analysis of clinical parameters and HDACsScore.

From the analysis mentioned above, we have identified 764 genes differentially expressed in three HDACsClusters. Based on these, univariate Cox analysis was performed, and 371 genes affecting prognosis were identified (Supplementary Table S2). The Lasso regression analysis was subsequently performed to avoid overfitting (Figures 4A,B). Filtered genes were included in the multivariate Cox regression analysis (stepwise regression) (Figure 4C), and we finally constructed a prognostic scoring system (we called HDACsScore), where a total of five genes (CRYM, GJB3, SLC2A1, STC1, and TUBB3) were included according to their risk coefficients (Figure 4D). Each patient was scored based on the following formula: HDACsScore = ExpressionCRYM * (−0.06596) + ExpressionGJB3 * (0.122546) + ExpressionSLC2A1 * (0.17376) + ExpressionSTC1 * (0.09823) + ExpressionTUBB3 * (0.14429). Based on the median HDACsScore, we divided patients into high- and low-HDACsScore groups. Survival analysis suggested a worse prognosis in patients in the high-HDACsScore group (Figure 4E; Supplementary Figure S7C). Figure 4F and Supplementary Figures S7D–E also visually showed high HDACsScore linking a poor prognosis. Subsequently, we explored the correlation between HDACsCluster and HDACsScore, and this result showed that HDACsCluster-B had the highest HDACsScore, followed by HDACsCluster-A (Figure 4G). From Figure 2B, we identified HDACsCluster-B had the worst prognosis, followed by HDACsCluster-A. This further supported the good performance of HDACsScore in patient prognostic stratification. We also applied an alluvial diagram including HDACsCluster, geneCluster, HDACsScore, and status to visualize the attribute changes of individual patients (Figure 4H). Further testing was performed to determine whether HDACsScore could independently predict patient outcome, and the results also confirmed that HDACsScore could be used as an independent predictor to assess patient prognosis (Figure 4I). Overall, this HDACsScore system could effectively identify patients with different prognoses and was expected to be extended to clinical practice.

### Genetic Features, Immune Infiltration, and Biological Processes of High- and Low-HDACsScore Groups

**FIGURE 5:**
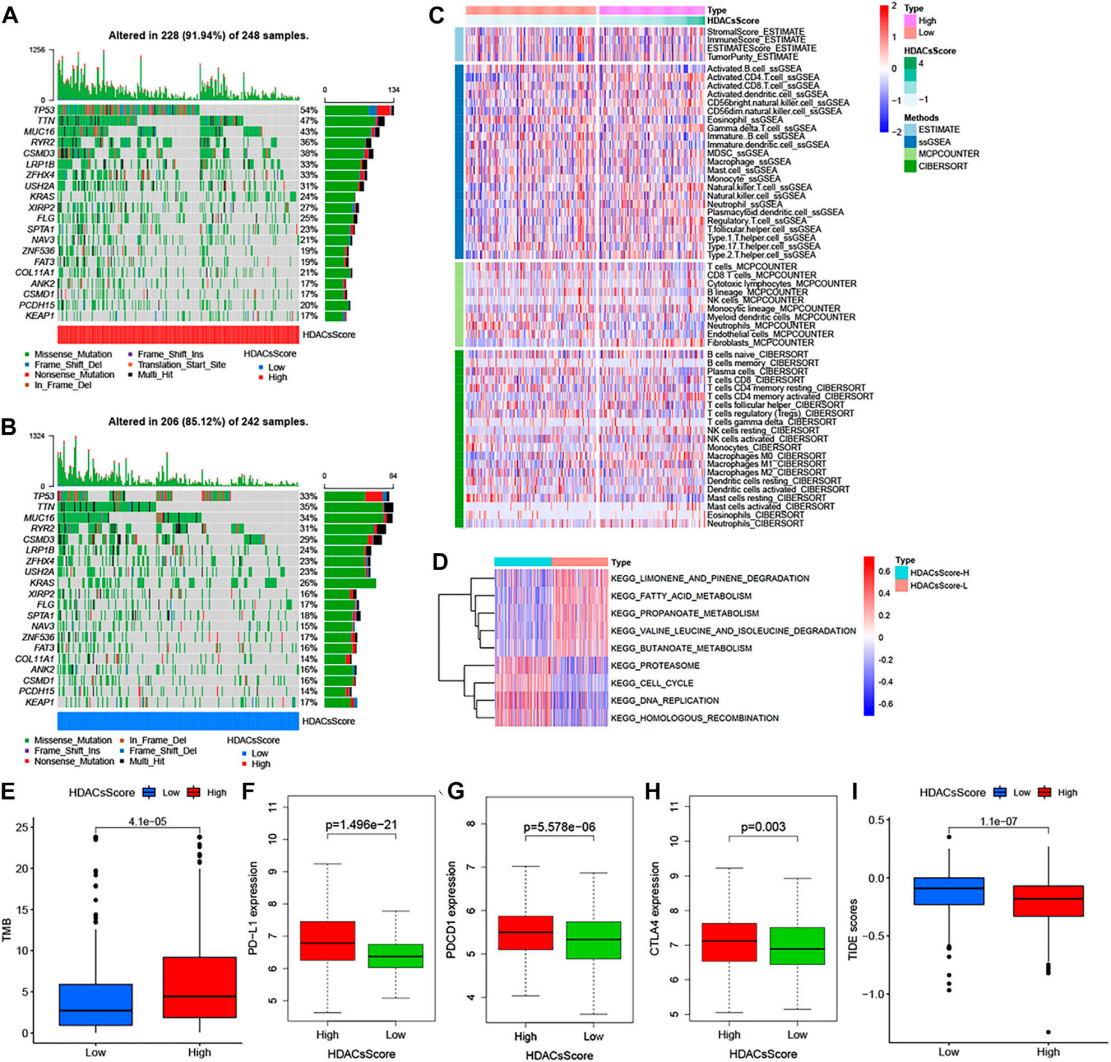
Genetic alteration landscape, immune infiltration, and biological characteristics in different HDACsScore subgroups. **(A–B)** Waterfall plot of tumor somatic mutation established in high- **(A)** and low- **(B)** HDACsScore groups. Each column represented individual patients. The upper barplot showed TMB, and the number on the right indicated the mutation frequency in each gene. The right barplot showed the proportion of each variant type. **(C)** Comparison of tumor immune infiltrating cells based on ssGSEA, CIBERSORT, and MCP counter algorithms in the high- and low-HDACsScore groups. Red indicated the high infiltrating levels of immune cells and blue indicated the low infiltrating levels. ESTIMATE score, stromal score, immune score, tumor purity, and subtype are also shown below the annotation. **(D)** GSVA enrichment analysis showing the activation states of biological pathways in high- and low-HDACsScore groups. The Wilcoxon rank sum test revealed significant difference on TMB **(E)**, expression of PD-L1 **(F)**, PDCD1 **(G)**, CTLA4 **(H)**, and TIDE score **(I)** between high- and low-HDACsScore groups.

The abovementioned analyses suggested significant differences in prognosis between high- and low-HDACsScore groups. In order to probe deeper into the potential genomic alterations, we then compared the somatic mutation landscapes in the two groups based on TCGA data. The high-HDACsScore group presented more extensive genetic mutation than the low-HDACsScore group (Figures 5A,B). Of the 20 genes with the highest mutation frequency, these genes all had higher mutation frequencies in the high-HDACsScore group of tumors than the low-HDACsScore group. In the high-HDACsScore group, TP53 presented a mutation frequency of up to 54% and tops the list. In the low-HDACsScore group, TIN had the highest frequency of mutations at 35%. Figure 5C depicted the immune infiltration landscapes with clear differences in high- and low-HDACsScore groups. To explore the biological behaviors between these two groups, we performed GSVA enrichment analysis. As shown in Figure 5D, the low-HDACsScore group was markedly enriched in body material metabolism processes, including fatty acid metabolism; valine, leucine, and isoleucine degradation; limonene and pinene degradation; propanoate metabolism; and butanoate metabolism, while the high-HDACsScore group highly enriched in homologous recombination, cell cycle, DNA replication etc. To sum up, these data enabled us to depict the impact of HDACsScore classification (high and low) on genomic variation, immune landscape, and biological pathways more comprehensively, where we found significant differences between high- and low-HDACsScore groups, which might be the intrinsic mechanism for the significantly different clinical outcomes between the two groups. Also, further investigation was still necessary.

### The Potential of HDACsScore in Predicting Immunotherapeutic and Chemotherapy Benefits

Accumulated evidence (Chan et al., 2019; Cristescu et al., 2018; Gong et al., 2018; Patel and Kurzrock, 2015) demonstrated patients with high-TMB status and high-immune checkpoint expression presented a durable clinical response to anti–PD-1/PD-L1 immunotherapy. In this study, the TMB quantification analyses confirmed that the high-HDACsScore group was markedly correlated with a higher TMB (Figure 5E), which was also able to be conjectured from Figures 5A,B. The Wilcoxon rank sum test revealed significant differences on expression of the immune checkpoints (PD-L1, PDCD1, and CTLA4) between low- and high-HDACsScore groups, and the high-HDACsScore group showed the higher immune checkpoint expression (Figures 5F–H). Based on the abovementioned findings, our preliminary inference was that the high-HDACsScore group might benefit more from immunotherapy. To further confirm this conjecture, we also compared the TIDE scores in the two groups. A lower TIDE score represented a higher response rate against both PD-1 and anti-CTLA-4 drugs. This result revealed that the TIDE score was remarkably decreased in the high-HDACsScore group (Figure 5I). This further suggested a higher sensitivity to immunotherapy in the high-HDACsScore group.

**FIGURE 6:**
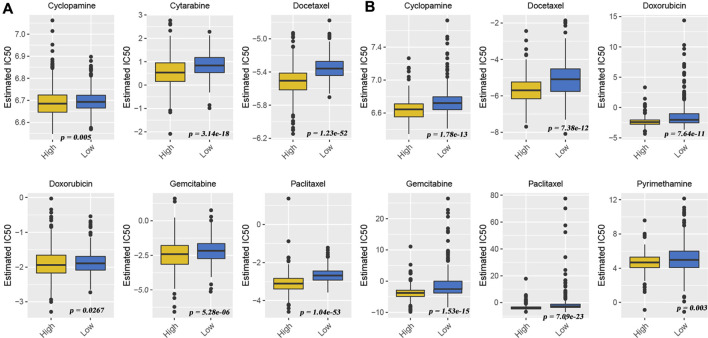
Drug sensitivity comparison between high- and low-HDACsScore groups. Distribution of the estimated IC_50_ of cyclopamine, docetaxel, doxorubicin, gemcitabine, paclitaxel, and pyrimethamine between high- and low-HDACsScore groups in the meta-GEO cohort **(A)** and TCGA cohort **(B)**.

Although many patients benefit from the rise of immunotherapy, there are still some patients who do not benefit from this advanced treatment. They had to return to traditional chemotherapy to prolong life. Effective identification of populations that may be sensitive to some type of chemotherapeutic agent is still being a matter of great significance. Therefore, we also used “pRRophetic” algorithms to compute the IC_50_ of some drugs in different patients to assess their sensitivity to a given drug. From the meta-GEO cohort (Figure 6A) and TCGA cohort data (Figure 6B), we found that the low-HDACsScore group had higher IC_50_ in six drugs (cyclopamine, docetaxel, doxorubicin, gemcitabine, paclitaxel, and pyrimethamine), indicating the patients in the high-HDACsScore group were more sensitive to these drugs. The abovementioned results initially illustrated the importance of HDACsScore in predicting the efficacy of immunotherapy and chemotherapy in LUAD. These results could provide more clues in determining the personalized treatment strategies for LUAD patients.

## Discussion

To our knowledge, this study is the only comprehensive analysis of HDAC family genes in LUAD. Herein, a systematic bioinformatics approach was used to comprehensively characterize the biological function, dysregulated expression, genomic mutations, immune infiltration, and prognostic relationships of HDAC genes in LUAD. Based on unsupervised clustering of HDACs, this study identified three different molecular subtypes mediated by HDACs, which presented different immune infiltration, prognoses, and transcriptome expression. The HDAC scoring system we constructed can effectively identify patients with different prognoses and make preliminary judgments on their response to chemotherapy and immunotherapy. High- and low-score groups represented different genomic landscapes, immune features, and signaling pathway activation states. This is of great significance for further exploring the prognostic heterogeneity as well as the potential targets of action in LUAD patients.

In this study, we observed mutations in the HDAC genes occurred in 15.15% of the LUAD samples, in which HDAC9 showed the highest mutation frequency (6%). Further investigation showed that this low-frequency mutation would promote HDAC2 expression and did not appear to affect the expression of other HDAC genes. HDAC9 was not differentially expressed in LUAD tumor tissues versus normal tissues; however, Kaplan−Meier analysis suggested that its overexpression was associated with better prognosis. A similar phenomenon was also observed in clear cell renal cell carcinoma (Fu et al., 2020) and retinoblastoma (Zhang et al., 2016). In other tumors, high expression of HDAC9 was usually associated with poor prognosis in patients, such as pancreatic cancer (Li et al., 2020), breast cancer (Huang et al., 2018), and oral squamous cancer (Rastogi et al., 2016). The abovementioned results indicated that HDAC9 had different prognostic implications in different tumors, and its influence on the tumor biological behavior may be influenced by the cancer context. Additionally, the correlation between the expression of HDAC family genes and immune cell infiltration remains poorly explored, and our study showed that most HDAC genes were positively correlated with each other, but negatively correlated with most immune cell infiltration. Among them, HDAC9 was negatively associated with all the other HDAC genes (such as HDAC10, HDAC11, SIRT2, SIRT3, SIRT4, SIRT5, and SIRT6), but positively correlated with most immune cell infiltration (activated B cell, activated CD4 T cell, activated CD8 T cell, activated dendritic cell, and NK cell). In the study of Ning et al. (2020), HDAC9 expression could modulate the tumor microenvironment, which in turn affected tumor biology behaviors. These results suggested that HDAC9 was closely related to tumor immunity, which could provide new horizons for in-depth relevant studies.

Significant heterogeneity brought great challenges to the prognostic judgment and treatment decisions in LUAD patients. Further identification of tumor subtypes with different characteristics facilitated the clinical adoption of precise strategies. The abovementioned analysis suggested the important roles of HDAC family genes in LUAD; hence, we believed that they probably played critical roles in the formation of different tumor subtypes and were implicated in cancer pathogenesis and progression. The three molecular subtypes identified in this study presented significantly different prognoses, and the expression of these 18 HDAC genes, biological pathway activation, and TME landscape across them also varied significantly. This may explain the internal mechanism of their different prognoses in the three molecular subtypes. We observed that compared with HDACsCluster-A and -B, HDACsCluster-C had the best survival but lowest immune checkpoint expression. This might somehow imply a poor response of HDACsCluster-C subtype to immunotherapy. To further explore the underlying molecular features and expression perturbations within these HDAC-mediated phenotypes, we performed a second unsupervised clustering and further determined that HDAC family genes indeed play an important role in LUAD prognosis, and the three subtypes identified in this study had good risk stratification performance. The construction of the HDAC prognostic scoring system aimed to eliminate prediction misjudgments caused by individual heterogeneity. Similar to previous prognostic systems (Li et al., 2021; Song et al., 2021; Zhang et al., 2020,^2021^), this HDAC prognostic scoring system accurately calculated the HDACsScore for each patient and then accurately predicts patient survival. We found that high HDACsScore was associated with poor prognosis, and HDACsScore could be used as an independent predictor to assess patient prognosis. In addition, we also observed that the high- and low-score groups were significantly different in somatic mutation characteristics, immune infiltration landscape, and biological pathway activation. Of the 20 genes with the highest mutation frequency, these genes changed more frequently in the high-HDACsScore group. In the high-HDACsScore group, TP53 mutation stated as high as 54%, topping the list. In the low-HDACsScore group, the TIN had the highest frequency of mutations at 35%. The material metabolic process was more active in the low-HDACsScore group, while the high-HDACsScore group was highly enriched in homologous recombination, cell cycle, and DNA replication. This seemed to explain why the high HDACsScore group presented a broader range of gene mutations.

Additionally, this study also investigated the potential of HDACsScore in predicting immunotherapeutic and chemotherapy benefits. It was shown that TMB status as well as immune checkpoint expression could suggest the tumor response to immunotherapy (Chan et al., 2019; Cristescu et al., 2018; Gong et al., 2018; Patel and Kurzrock., 2015). In our study, TMB quantification analyses confirmed that the high-HDACsScore group was markedly correlated with higher TMB. Also, the high-HDACsScore group also showed higher immune checkpoint expression. Furthermore, TIDE analysis also yielded consistent results. The abovementioned findings suggested a higher sensitivity to immunotherapy in the high-HDACsScore group. In addition, the IC_50_ of a given drug could reflect its sensitivity to the drug. This study found that the low-HDACsScore group had higher IC_50_ in cyclopamine, docetaxel, doxorubicin, gemcitabine, paclitaxel, and pyrimethamine, indicating patients in the high-HDACsScore group were more sensitive to these drugs. Overall, the abovementioned results initially illustrated the importance of HDACsScore in predicting the efficacy of immunotherapy and chemotherapy in LUAD. These results could provide more clues in determining the personalized treatment strategies for LUAD patients.

This study, based on a large LUAD cohort including seven independent datasets, revealed the potential significance of HDAC family genes in LUAD. However, there were still some limitations in this study. Although we used the “ComBat” algorithm of the “sva” package in R to further integrate into a metacohort, batch effects resulting from nonbiotechnological bias could not be completely eliminated. It was still difficult to avoid the bias imposed by the nature of the retrospective study. In addition, this study lacked extensive experimental validation with results derived from public database. Overall, the present study comprehensively evaluated the potential significance of HDAC family genes in over 1400 LUAD samples and described the multidimensional characterization of HDAC family genes in LUAD. More broadly, based on the HDAC genes, this study identified three LUAD subtypes with different genomic, transcriptome, immune infiltration, and metabolic pathway characteristics and constructed an HDAC scoring system for risk stratification and efficacy prediction, which would help enhance our perception of LUAD prognostic differences and provide important insights into the efficacy of immunotherapy and chemotherapy.

## Data Availability

The datasets presented in this study can be found in online repositories. The names of the repository/repositories and accession number(s) can be found in the article/Supplementary Material.
