# Supplementary figures and images for: Integrated Analysis Reveals the Potential Significance of HDAC Family Genes in Lung Adenocarcinoma
Source: Front Genet. 2022 Aug 22;13:862977. doi: 10.3389/fgene.2022.862977 (PMC9441483; doi:10.3389/fgene.2022.862977)

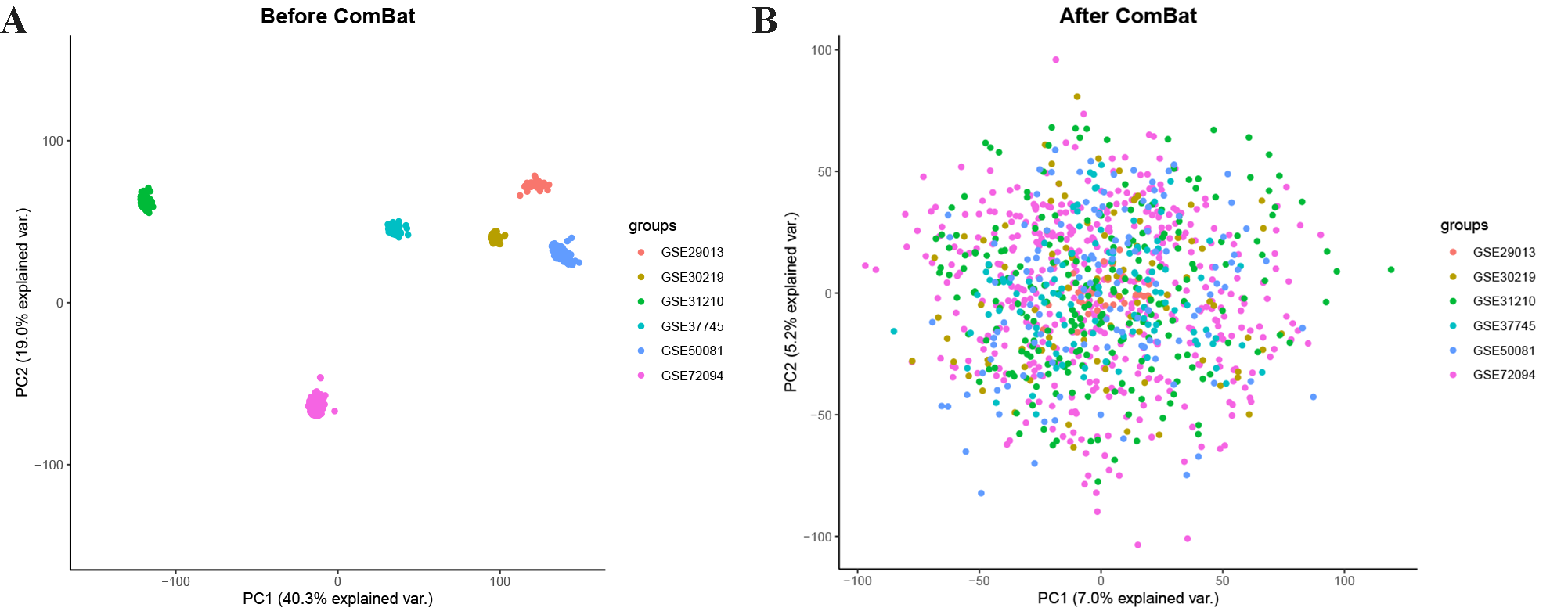

Supplement: Supplementary file 2 [file Image6.TIF]

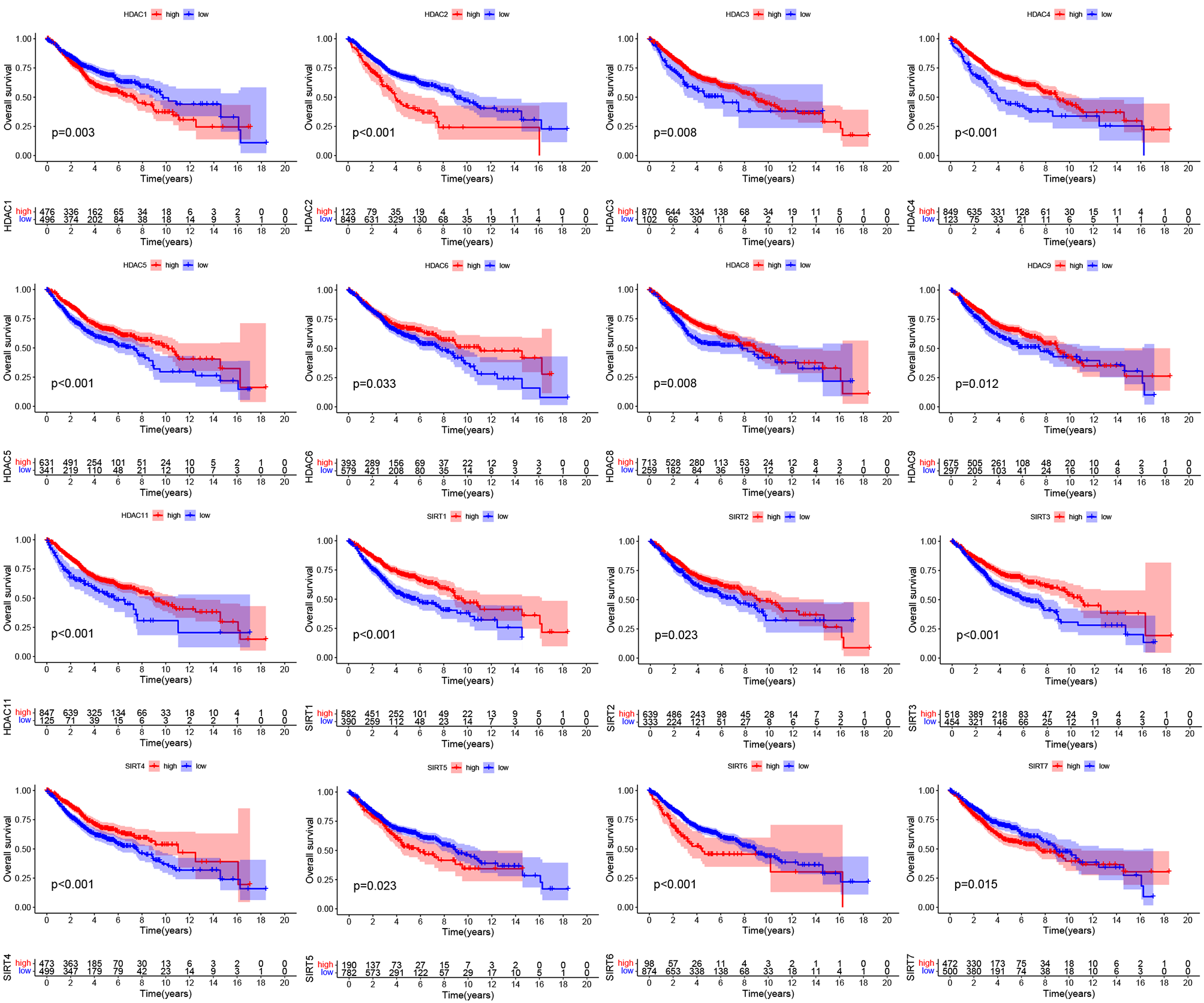

Supplement: Supplementary file 3 [file Image3.TIF]

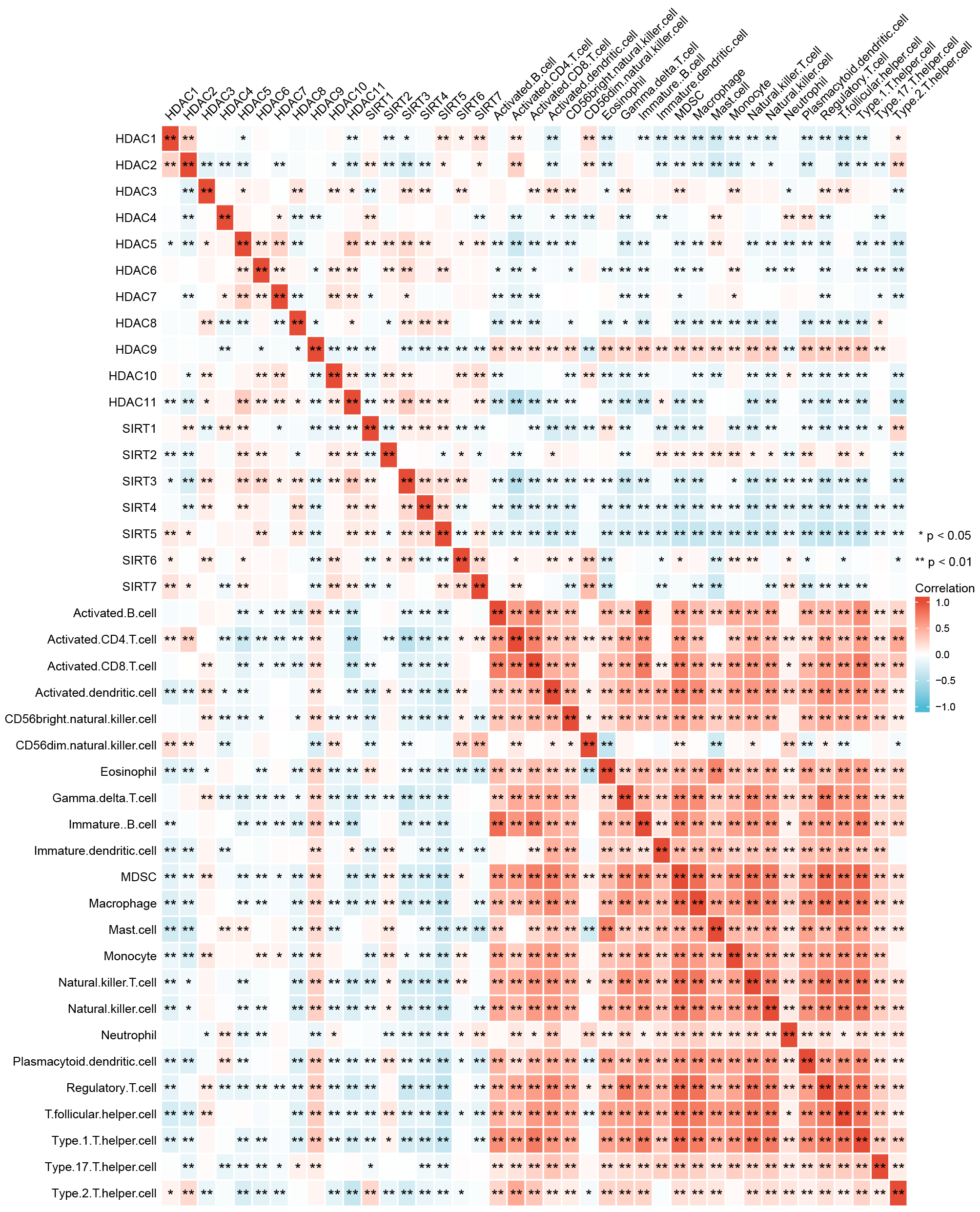

Supplement: Supplementary file 4 [file Image4.TIF]

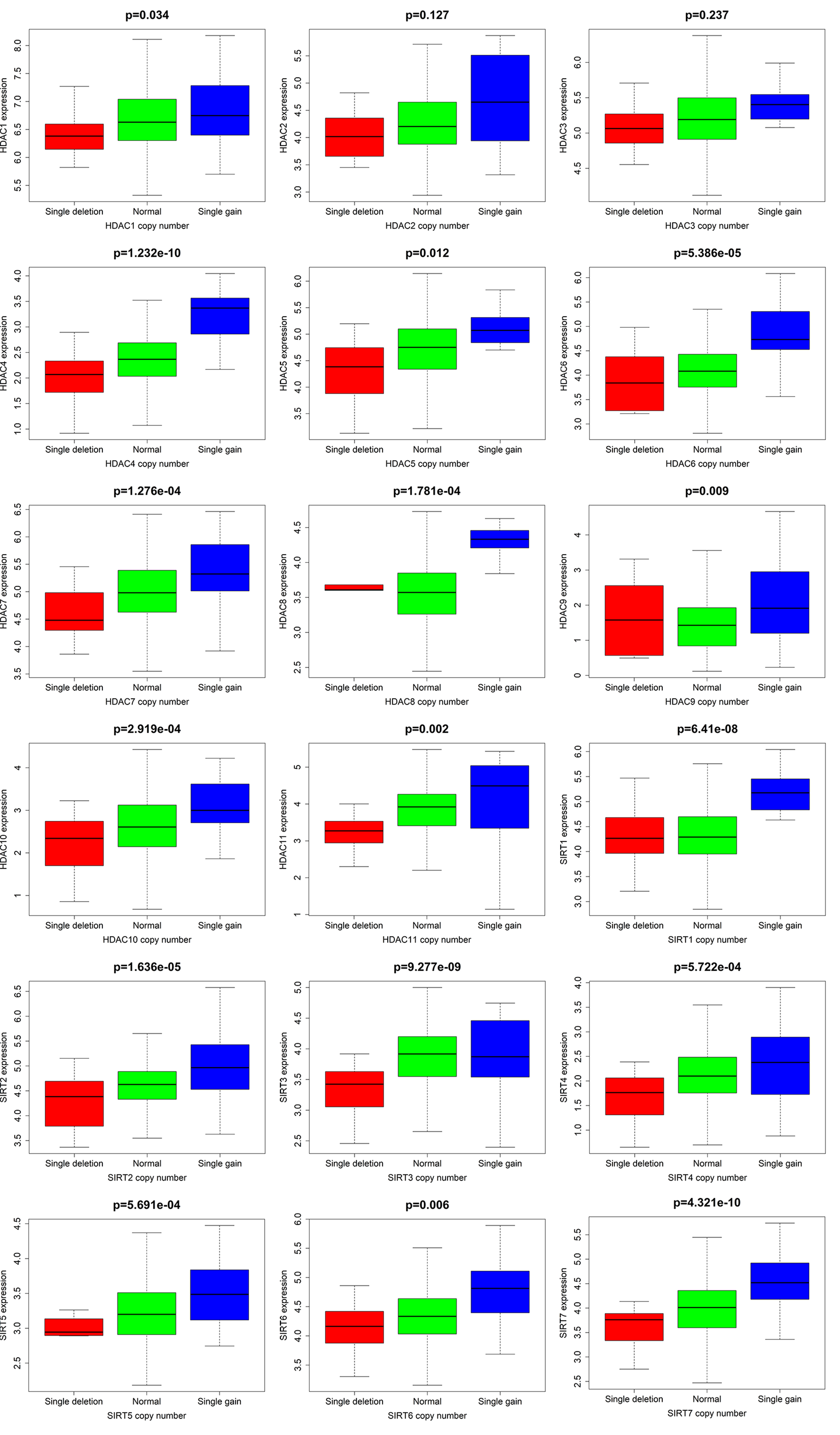

Supplement: Supplementary file 5 [file Image2.TIF]

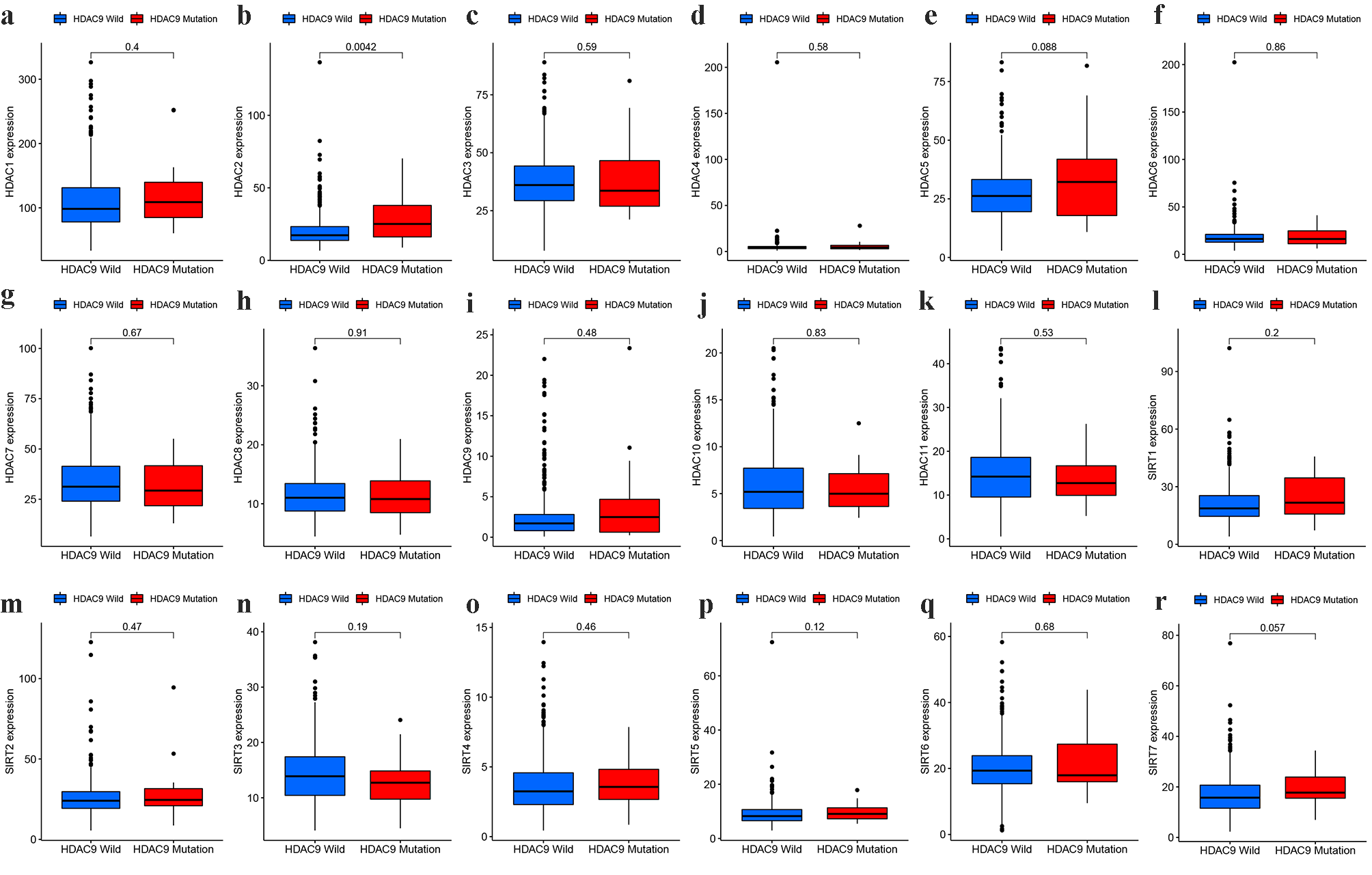

Supplement: Supplementary file 6 [file Image1.TIF]

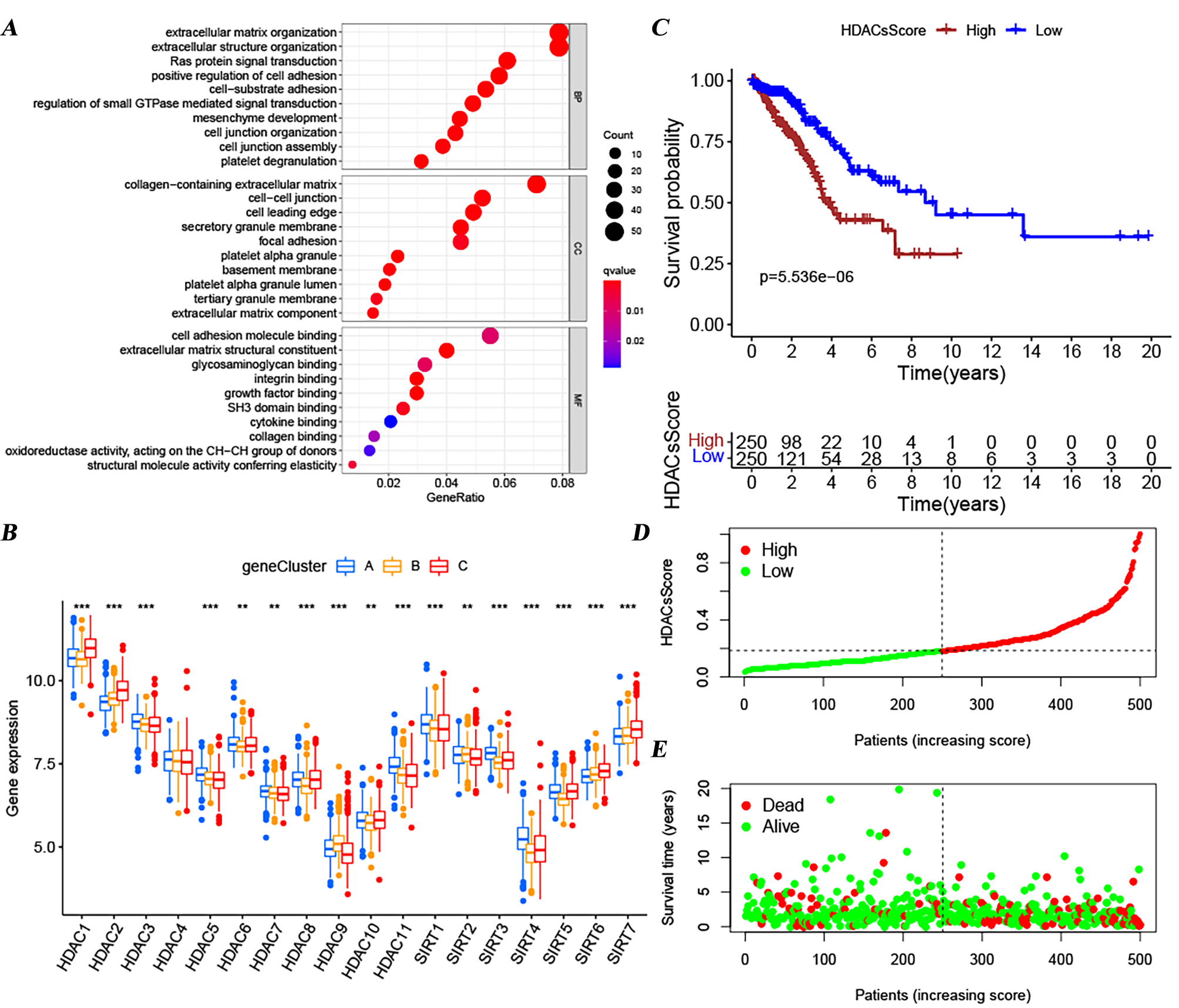

Supplement: Supplementary file 7 [file Image7.TIF]

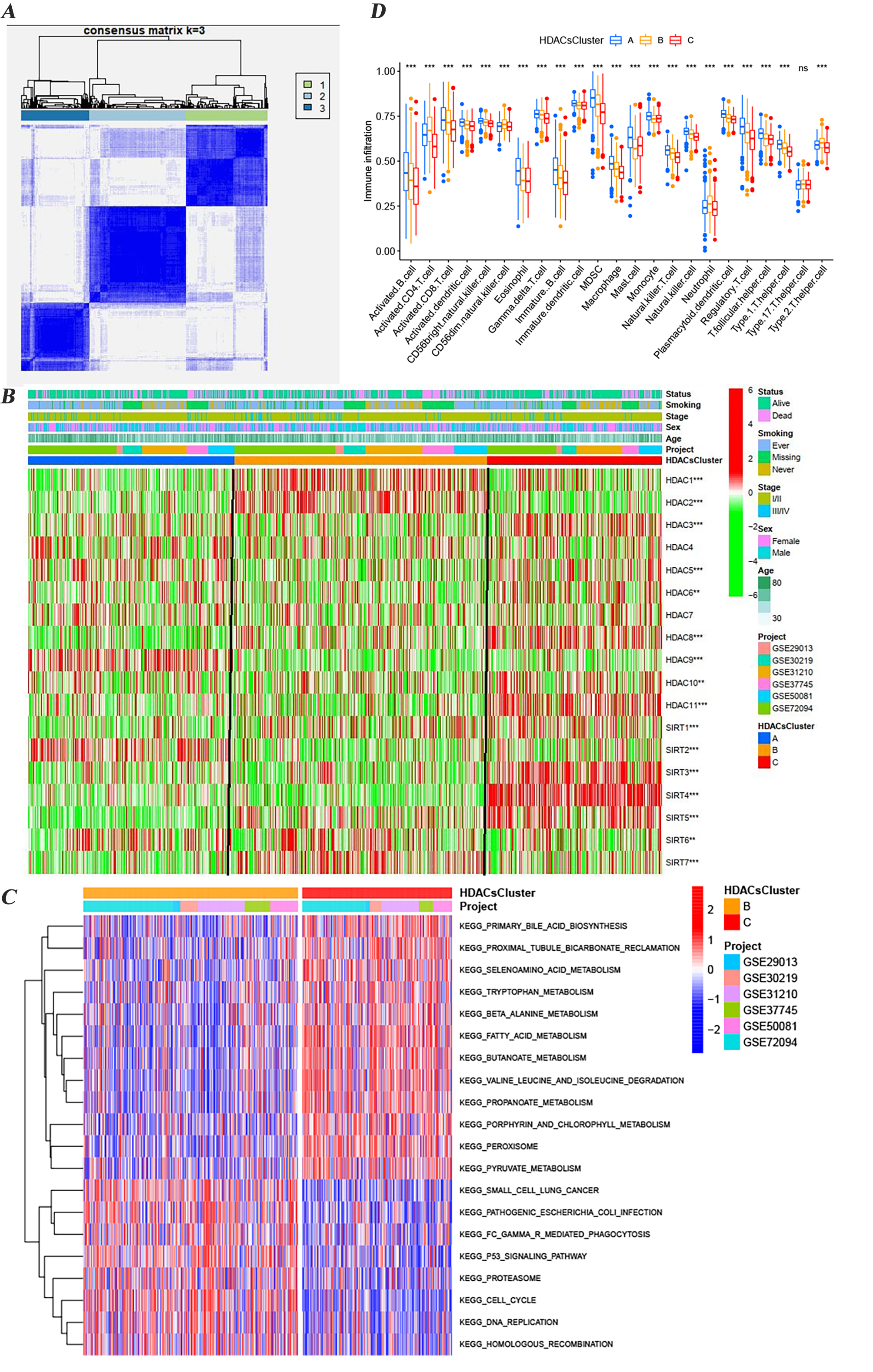

Supplement: Supplementary file 9 [file Image5.TIF]
